# 24-epibrassinolide restores nitrogen metabolism of pigeon pea under saline stress

**DOI:** 10.1186/1999-3110-54-9

**Published:** 2013-08-21

**Authors:** Ronaldo José Durigan Dalio, Hildete Prisco Pinheiro, Ladaslav Sodek, Claudia Regina Baptista Haddad

**Affiliations:** 1 Department of Plant Biology, Institute of Biology, State University of Campinas-UNICAMP, CP 6109 Campinas, SP 13083-970 Brazil; 2 Department of Ecophysiology of Plants, Technical University of Munich, Hanz-Carl-von-Carlowitz-Platz 2, 85354 Freising, Germany; 3 Departament of Statistics, Institute of Mathematics, Statistics and Scientific Computation, University of Campinas-UNICAMP, Campinas, SP 13083-970 Brazil

**Keywords:** *Cajanus cajan*, Brassinosteroid synthesis inhibitor, Nitrate uptake, Nitrogen metabolism, N-transport amino acids, Saline stress, Xylem amino acids, Xylem exudate

## Abstract

**Background:**

Several studies have shown that brassinosteroids attenuate the effects of salt stress. However, nothing is known about their effects on amino acid transport, nor the effects of these hormones on nitrate uptake under saline conditions. This study set out to determine the effects of 24-epibrassinolide, at concentrations of 10-7 M and 0.5 × 10-9 M, and clotrimazole (inhibitor of brassinosteroid synthesis), at 10-4 M, on nitrate uptake and metabolism in plants of *C. cajan* (L.) Millsp, cultivar C11, growing under salinity. The following aspects were analyzed: levels of proteins, amino acids, nitrate, nitrate reductase of roots and the composition of xylem sap amino acids.

**Results:**

Salinity reduced the proportion of N-transport amino acids ASN (the major component), GLU, ASP and GLN. The effect of the hormone in reducing the adverse effects of salt was related to the reestablishment (totally or partially) of the proportions of GLU, ASN and GLN, transported in the xylem and to the small but significant increase in uptake of nitrate. Increased nitrate uptake, induced by 24- epibrassinolide, was associated with a higher activity of nitrate reductase together with greater levels of free amino acids and soluble proteins in roots of plants cultivated under saline conditions.

**Conclusion:**

The decline in several components of nitrogen metabolism, induced by salt, was attenuated by 24-epibrassinolide application and accentuated by clotrimazole, indicating the importance of brassinosteroid synthesis for plants growing under salinity.

**Electronic supplementary material:**

The online version of this article (doi:10.1186/1999-3110-54-9) contains supplementary material, which is available to authorized users.

## Background

Brazil has around 90 million hectares of salinized soils (FAO 2008). Salt stress adversely affects the development of plants (Zheng et al. 2008; Türkan and Demiral 2009 Galvan-Ampudia and Testerink 2011). Among the adverse effects of salts are the altered uptake and transport of nitrate (Rubinigg et al. 2003; Parida and Das 2004; Carillo et al. 2005; Maaroufi-Dguimi et al. 2011). Reduced nitrate uptake affects nitrogen assimilation (Silveira et al. 2001; Qu et al. 2011), with consequent alterations in amino acid and protein metabolism (Surabhi et al. 2008; Younis et al. 2009; Maaroufi-Dguimi et al. 2011). Nevertheless, very few studies (Cramer et al. 2002) have investigated changes in amino acid transport to the shoot via xylem under these conditions. Not only may such changes be a useful indicator of stress (Marsh and Adams 1995; Cramer et al. 2002; Amarante et al. 2006; Renault et al. 2010) but they can reflect dynamic aspects of amino acid metabolism occurring in the root, often not apparent from analyses of amino acids of the source organ (Amarante and Sodek 2006).

Since the recognition of brassinosteroids as growth regulators, many studies have been carried out concerning their capacity of attenuating the effects of diverse types of stress, including salt stress (Zullo and Adam 2002; Ozdemir et al. 2004; Kagale et al. 2007). Despite such reports and the well documented adverse effects of salt on N metabolism, information is scarce (Anuradha and Rao 2001) regarding the influence of these hormones on nitrate uptake and metabolism under saline conditions.

*Cajanus cajan* (L.) Millsp. is a legume cultivated in the tropics and sub-tropics (Summerfield and Roberts, 1985 apud Akintayo et al. 1999). Their seeds are consumed as a supplement in flour or *in natura* (Corzo and Fuentes 2004). In a previous study (Dalio et al. 2011) 24-epibrassinolid reduced the inhibitory effects of NaCl on photosynthesis and several growth parameters of *C. cajan* plants. In this study, we investigated whether 24-epibrassinolide can revert the adverse effects of salt on growth of *C. cajan* through changes in nitrate uptake and metabolism. The effect of clotrimazole, an inhibitor of brassinosteroid synthesis (Amzallag and Vaisman 2006), was also studied. Although our study focused on N metabolism in the root (in contrast to many studies) we included the analysis of amino acids in the xylem sap in view of the absence of information regarding the influence of brassinosteroids on nitrogen transport to the shoot.

## Methods

The study was carried out in Campinas, São Paulo State, Brazil (22°49′ S, 47°06′ W, and 670 m of altitude). Seeds of *Cajanus cajan* (L.) Millsp, cultivar C11, supplied by the Agronomy Institute at Campinas, were allowed to imbibe in distilled water for 48 hours and then sown in 700 mL plastic pots, containing perlite. The plants were grown in the greenhouse for approximately 50 days, under natural light and temperature (19 to 40°C) conditions, from February to April of 2007. The pots were watered daily with tap water until the fall of the cotyledons. At this stage each pot, containing a single plant, received 150 mL of Hoagland and Arnon’s (1938) normal-strength nutrient solution, with or without added NaCl.

During the exposure of plants to saline stress, the plants were given gradually increasing concentrations of salt, in order to avoid osmotic shock. The saline concentrations were increased by 50 mM every two days, until the final concentration of 400 mM were reached. In all, the plants were exposed to salt for 16 days.

In order to guarantee uniform exposure of the roots to the salt, the pots (with holes in the base) were placed inside a second pot of greater diameter with no holes containing excess nutrient solution (to a depth of about 1 cm). This ensured that the nutrient solution kept the perlite moist up to the surface by capillary action. Before each application of nutrient solution, the outer pot was removed and the inner pots washed with 150 mL of nutrient solution, in order to avoid salt accumulation. The pots were allowed to drain for several hours, so as to avoid dilution of the subsequently added salt/nutrient solution.

The brassinosteroid 24-epibrassinolide (Sigma-E1641) was applied to the substrate one week before the application of salt. The hormone was dissolved in ethanol (1 mg mL^-1^) and diluted in nutrient solution to final concentrations of 10^-7^ M and 0.5 × 10^-9^ M.

The inhibitor of steroid synthesis Clotrimazole (Sigma C6019) was first dissolved in DMSO (dimethylsulfoxide) and then added to the nutrient solution (in the proportion of 1 mL of DMSO L^-1^ of solution) to give a concentration of 10^-4^ M and applied to the substrate one week before the application of the salt.

The roots were harvested under running water: one part was used for measuring nitrate reductase activity and the rest for extraction of metabolites. The latter portion of roots was frozen, lyophilized and ground in a mortar. The powder was extracted with methanol: chloroform: water (MCW – 12:5:3 v/v) (Bieleski and Turner 1966). The extract was kept at 5°C for 24 hours and then centrifuged at 2000 *g* (Fanem, 215, Brazil), for 30 minutes. The supernatant was collected and, for each 4 mL, 1 mL of chloroform and 1.5 mL of water were added followed by vigorous shaking. After standing for 24 hours, the chloroform phase was discarded and the aqueous phase left in a water bath at 38°C, for 8 hours, to eliminate residual chloroform and concentrate the samples. Then, the samples were centrifuged at 14000 *g* (Beckmann, Avanti J30 I, USA) for 5 minutes, and the supernatant collected. Samples were stored at −20°C (Operon Co. Ltd, Korea) and used for the determination of nitrate and free amino acids. The residue after MCW extraction was retained for the determination of root protein content.

To evaluate the concentration of proteins the residue after MCW extraction was suspended in 10 mL of 0.1 N NaOH and homogenized with a glass rod. After 24 hours of extraction the suspension was centrifuged at 2000 *g* for 30 minutes. The supernatant was used for the determination of protein as described by Bradford (1976), using bovine serum albumin as standard.

Nitrate determination followed the method of Cataldo et al. (1975) using KNO_3_ as standard and free amino acids were determined according to Yemm and Cocking (1955), using leucine as standard.

Nitrate reductase was extracted and assayed essentially as described by Botrel and Kaiser (1997). Nitrite in the assay was determined on aliquots removed at zero time and after 30 minutes incubation according to Hageman and Reed (1980).

The xylem bleeding sap was collected according to Mc Clure and Israel (1979). The stem was severed with a razor blade just below the cotyledonary node and the exudate collected with microcapillaries. Sap samples were stored frozen until further analysis.

Amino acid composition of xylem sap was determined by reverse phase HPLC (LKB 2150, Spectra Physics Ltd, USA) of the o-phthaldialdehyde (OPA) derivatives based on the method of Jarret et al. (1986), with modifications (Thomas et al. 2005).

The trials were set up in a completely randomized design. One concentration of NaCl was used at 400 mM together with a control (NaCl absent). Plants were cultivated with and without previous application of 24-epibrassinolide (two concentrations) or clotrimazole, all in the presence and absence of salt, giving a total of 8 treatments. The treatments had 12 replicates, where each replicate consisted of a single pot with one plant. For the analysis of amino acid composition four replicates were used each consisting of material from three plants. The data were submitted to an analysis of variance (ANOVA) for three factors (salt, concentration of hormone and of clotrimazole). When significant interaction occurred the analysis of variance was repeated considering each treatment separately (One-Way ANOVA) and the means compared by Duncan’s multiple range test (p ≤ 0.05).

## Results

Significant interaction was found between the three factors under study (salt, hormone and clotrizamole), for the various parameters evaluated. Each combination of variables was therefore analyzed separately.

**Figure 1:**
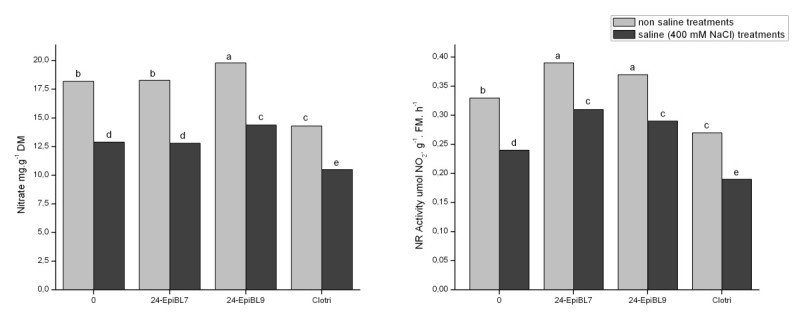
**Effect of 24-epibrassinolide, at 10**^**-7**^ **M (EpiBL7) or 0.5 × 10**^**-9**^ **M (EpiBL9) and of the inhibitor of brassinosteroid synthesis (Clotri) on nitrate levels and nitrate reductase activity (NR) in roots of** ***Cajanus cajan*** **, cultivated without or with 400 mM NaCl.** Means followed by different letters are different (Duncan, *p* < 0.05). DM= dry mass.

**Figure 2:**
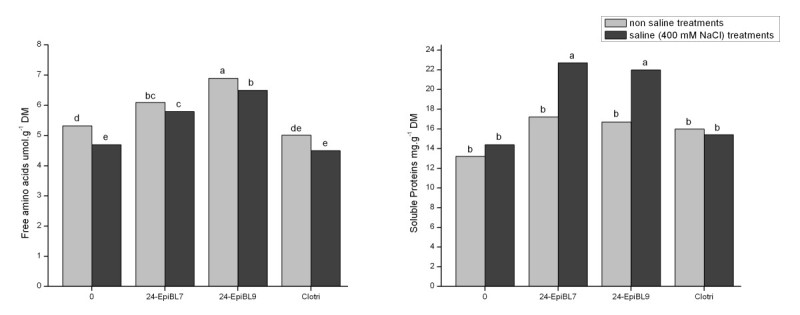
**Effect of 24-epibrassinolide, at 10**^**-7**^ **M (EpiBL7) or 0.5 × 10**^**-9**^ **M (EpiBL9) and of the inhibitor of brassinosteroid synthesis (Clotri) on free amino acid and soluble protein levels in roots of** ***Cajanus cajan*** **, cultivated without or with 400 mM NaCl.** Means followed by different letters are different (Duncan, *p* < 0.05). DM= dry mass.

The effect of salt on the levels of nitrate and nitrate reductase activity in roots of *C. cajan* plants are shown in Figure 1 and on the levels of free amino acids and soluble proteins in Figure 2. With the exception of soluble proteins, there were reductions in all parameters analysed.

In the absence of saline stress, applications of 24-epibrassinolide led to small but significant increases in nitrate reductase activity as well as of nitrate (except at 10^-7^ M) and amino acids whereas clotrimazole resulted in reduced levels of nitrate and nitrate reductase activity (Figures 1 and 2).

If only plants cultivated under salinity are compared, it may be seen that applications of 24-epibrassinolide resulted in increases in all parameters of nitrogen metabolism measured while the inhibitor of these hormones lead to a reduction in nitrate and nitrate reductase activity, but not free amino acids or soluble proteins (Figures 1 and 2).

**Figure 3:**
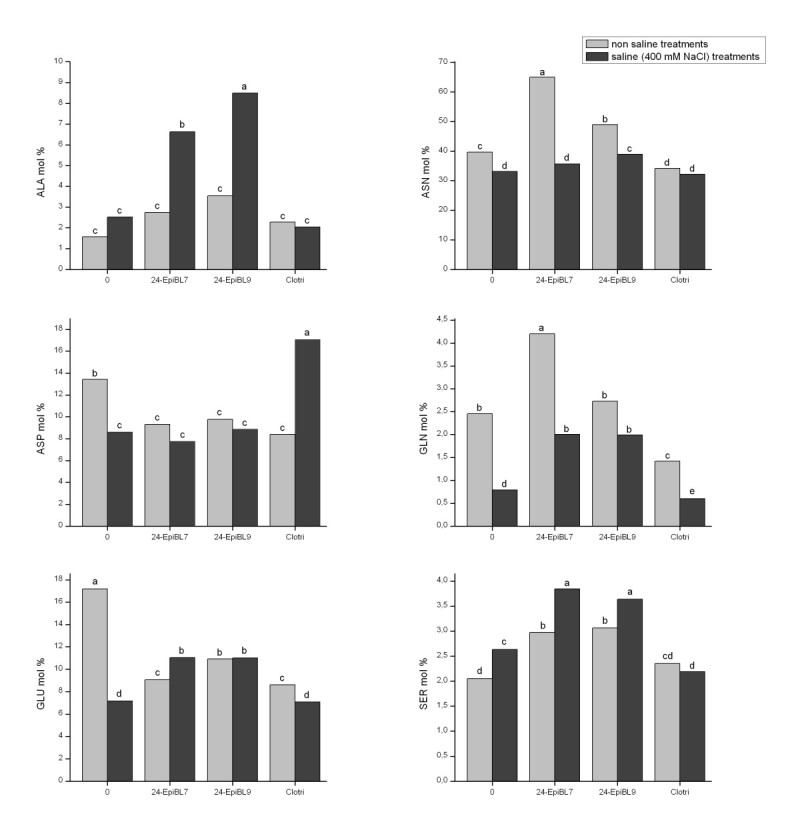
**Effect of 24-epibrassinolide, at 10**^**-7**^ **M (EpiBL7) or 0.5 × 10**^**-9**^ **M (EpiBL9) and of the inhibitor of brassinosteroid synthesis (Clotri) on free amino acid levels in the xylem sap of** ***Cajanus cajan*** **, cultivated without or with 400 mM NaCl.** Means followed by different letters are different (Duncan, *p* < 0.05). The following amino acids were also detected: HIS, GLY, THR, ARG, TYR, MET, VAL, PHE, ILE, LEU, LYS, GABA however, none were present at values greater than 3%.

The effects of salinity and the 24-epibrassinolide on amino acid transport in the xylem sap of *C. cajan* plants may be seen in Figure 3, where data are shown for the more important amino acids (GLN, GLU, ASN, ASP, ALA), that is, those most closely associated with primary nitrogen assimilation and often prominent in nitrogen transport. SER was also included since it often follows changes in ALA associated with some stress conditions (Sousa and Sodek 2003). The amino acids transported in greatest amounts were ASN (40% in the control) followed by GLU and ASP (17% and 13% respectively).

In general, under salt treatment or after the application of clotrimazole, it may be seen that the proportion of the three prominent amino acids (ASN, ASP, GLU) declined relative to the control. On the other hand, ALA and SER increased in most of the saline treatments, with and without hormone application. The only exception was ALA in plants cultivated under salinity and without hormone which did not differ from the control plants.

With the application of 10^-7^ M 24-epibrassinolide in the absence of salt, the proportion of ASN increased to 65% of the amino acids transported in the xylem sap and in the treatment with the lower concentration of hormone (24-epibrassinolide at 0.5 × 10^-9^ M ) the proportion of this amino acid reached approximately 50%. This increase was accompanied by a decline in GLU and ASP. Clotrizamole, in the absence of salt, caused the opposite effect, a decline in ASN.

Under salt treatment it may be seen that the proportion of the three prominent amino acids (ASN, ASP, GLU) declined relative to the control, while ALA did not decline and SER increased under salt treatment. Application of 24-epibrassinolide to plants subjected to salt stress resulted in increases in proportions of the following amino acids in relation to the saline control: ALA, SER, GLN and GLU, and, at the lower hormone concentration at least, ASN. ASP, however, remained at a level similar to the salt control. These increases were not seen with the application of clotrimazole, while ASP showed an increase in contrast to the hormone treatment where it remained at the level of the salt control.

## Discussion

The reduced level of root nitrate under saline conditions observed in this study is consistent with that observed for seedlings or leaves of other species (Silveira et al. 2003; Debouba et al. 2007), when cultivated under saline conditions. It is to be expected that reduced uptake of nitrate would lead to lower activity of nitrate reductase, as has been observed for other species (Hamdia et al. 2004; Iqbal et al. 2006; Debouba et al. 2007; Hasaneen et al. 2008), although only the first study included data for roots as in our study*.* Probably, the close relationship between nitrate assimilation and amino acid biosynthesis explains the reduced levels of free amino acids found in roots of *C. cajan* plants after salt treatment. In contrast to amino acids, the levels of soluble proteins were not affected by salinity. Stability of protein levels in *C. cajan* plants under saline stress was also observed by Ashraf (1994). Possibly, the maintenance of soluble protein levels reflects an increase in stress-specific proteins (Younis et al. 2009) that compensate a decline in other proteins.

The positive effect of 24-epibrassinolide with some parameters did not occur exclusively in the presence of salt, indicating that the hormonal effect was not necessarily salt specific. However, the effect of the hormone evidently has greater importance in the presence of salt stress. The positive effect of 24-epibrassinolide on nitrate levels for the *C. cajan* plants cultivated under salinity indicate increased uptake, leading to higher nitrate reductase activity, which in turn could explain the higher levels of free amino acids and soluble proteins. An increase in nitrate reductase activity was also reported by Anuradha and Rao (2001) following application of 24-epibrassinolide in rice plants subjected to saline conditions. Possibly, brassinosteroids maintain the integrity of the cell membrane under stress conditions and consequently preserve the process of nitrate uptake. Hayat et al. (2007) found that 28-homobrassinolide reduced the inhibitory effect of cadmium on nitrate uptake of *Brassica juncea* and attributed the inhibitor effect of the metal to possible damage to the cell membranes.

Studies of amino acid export in the xylem under saline conditions are scarce in the literature (Cramer et al. 2002). Analysing changes in the amino acid composition of the xylem sap can be a powerful tool, since they can reflect changes in metabolic processes occurring in the source organs (Amarante and Sodek 2006). In this study ASN was the most abundant amino acid in the xylem sap, a general characteristic of legumes (Lea et al. 2007). Our data show diminished ASN in the xylem sap under saline stress, together with ASP, GLU and GLN. A reduction in the proportion of ASN in the root bleeding sap of *Casuarina glauca* under saline stress has also been reported (Cramer et al. 2002). Reduced xylem sap ASN can be attributed to diminished activity of asparagine synthetase in the root system (Lima and Sodek 2003; Antunes et al. 2008).

The reduction in the proportion of the amino acids ASN and GLN due to salinity was totally and GLU partially reverted by the application of 24-epibrassinolide, since the composition returned to values equal or closer to those seen for the controls without salt. Applications of the inhibitor of brassinosteroid synthesis resulted in reductions in the proportions of these amino acids in relation to the control in both the presence and absence of salt, reinforcing the idea that 24-epibrassinolide is associated with the increase in these amino acids in the xylem. We are unaware of any report concerning a possible mechanism of action of brassinosteroids in relation to the synthesis and transport of amino acids. However, it does seem that the hormone is involved in such processes, since the application of 24-epibrassinolide re-established, at least partially, the proportions of these amino acids.

The strong positive effect of 24-epibrassinolide on ALA in the presence of salt (and to a lesser extent on SER) was not expected in view of the association of increased ALA (and SER) with abiotic stress (Puiatti and Sodek 1999; Sousa and Sodek 2002; Yazici et al. 2007). The hormone in the absence of salt and salt alone did promote small increases in these two amino acids, but the increases in ALA were not significant, suggesting a very weak association with salt stress. Perhaps in this case the increases in ALA and SER promoted by the hormone in the presence of salt were not related to a stress response. Rather, it would appear that salt does not interfere with the positive effect of the hormone on these two amino acids.

## Conclusion

In conclusion, the positive effect of 24-epibrassinolide on nitrate uptake under salinity was reflected in the increased activity of nitrate reductase in the root, that probably underlies the higher levels of free amino acids and soluble proteins found in these organs. Application of the hormone also fully or partially restored the proportions of amino acids ASN, GLN and GLU, the products of primary nitrate diminished in the xylem sap by salinity. Application of the brassinosteroid synthesis inhibitor caused a decrease in the proportions of these amino acids and intensified the adverse effects of salinity on nitrate concentration and activity of nitrate reductase*.* Overall, the hormone exerts a beneficial effect on nitrogen metabolism in roots of *C. cajan* plants under saline stress and its mode of action would appear, therefore, to involve the restoration of N metabolism close to that found in unstressed plants.

## Authors’ original submitted files for images

Below are the links to the authors’ original submitted files for images. Authors’ original file for figure 1 Authors’ original file for figure 2 Authors’ original file for figure 3

## Abbreviations

ASN: Asparagine
ASP: Aspartic acid
GLN: Glutamine
GLU: Glutamic acid.
