# Supplementary figures and images for: 24-epibrassinolide restores nitrogen metabolism of pigeon pea under saline stress
Source: Bot Stud. 2013 Aug 21;54:9. doi: 10.1186/1999-3110-54-9 (PMC5430367; doi:10.1186/1999-3110-54-9)

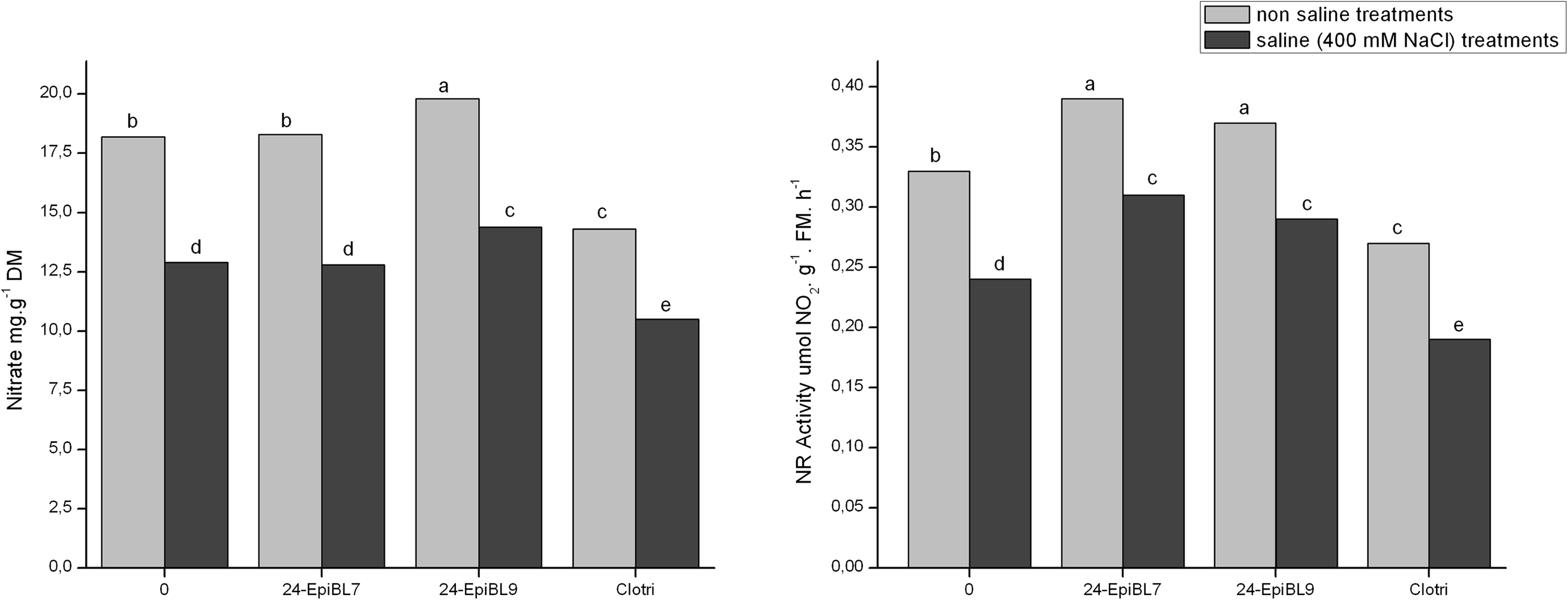

Supplement: Supplementary file 1 — Authors’ original file for figure 1 [file 40529_2012_8_MOESM1_ESM.tif]

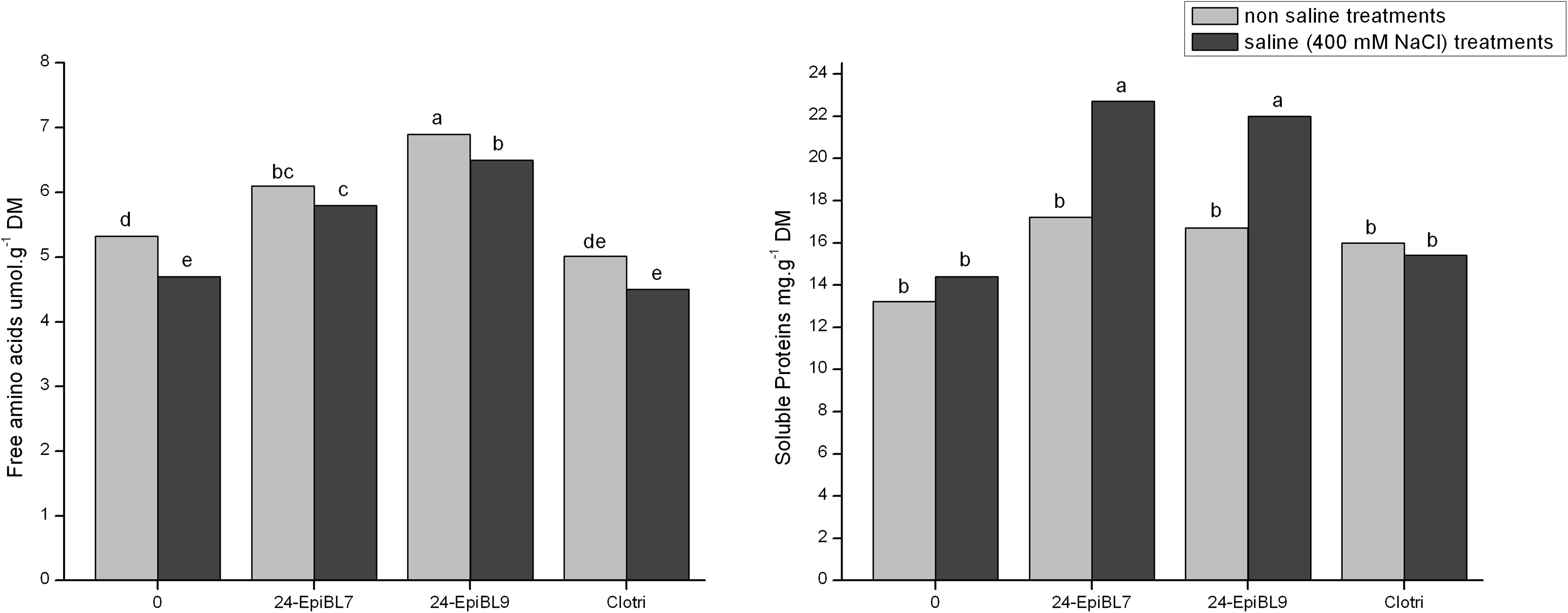

Supplement: Supplementary file 2 — Authors’ original file for figure 2 [file 40529_2012_8_MOESM2_ESM.tif]

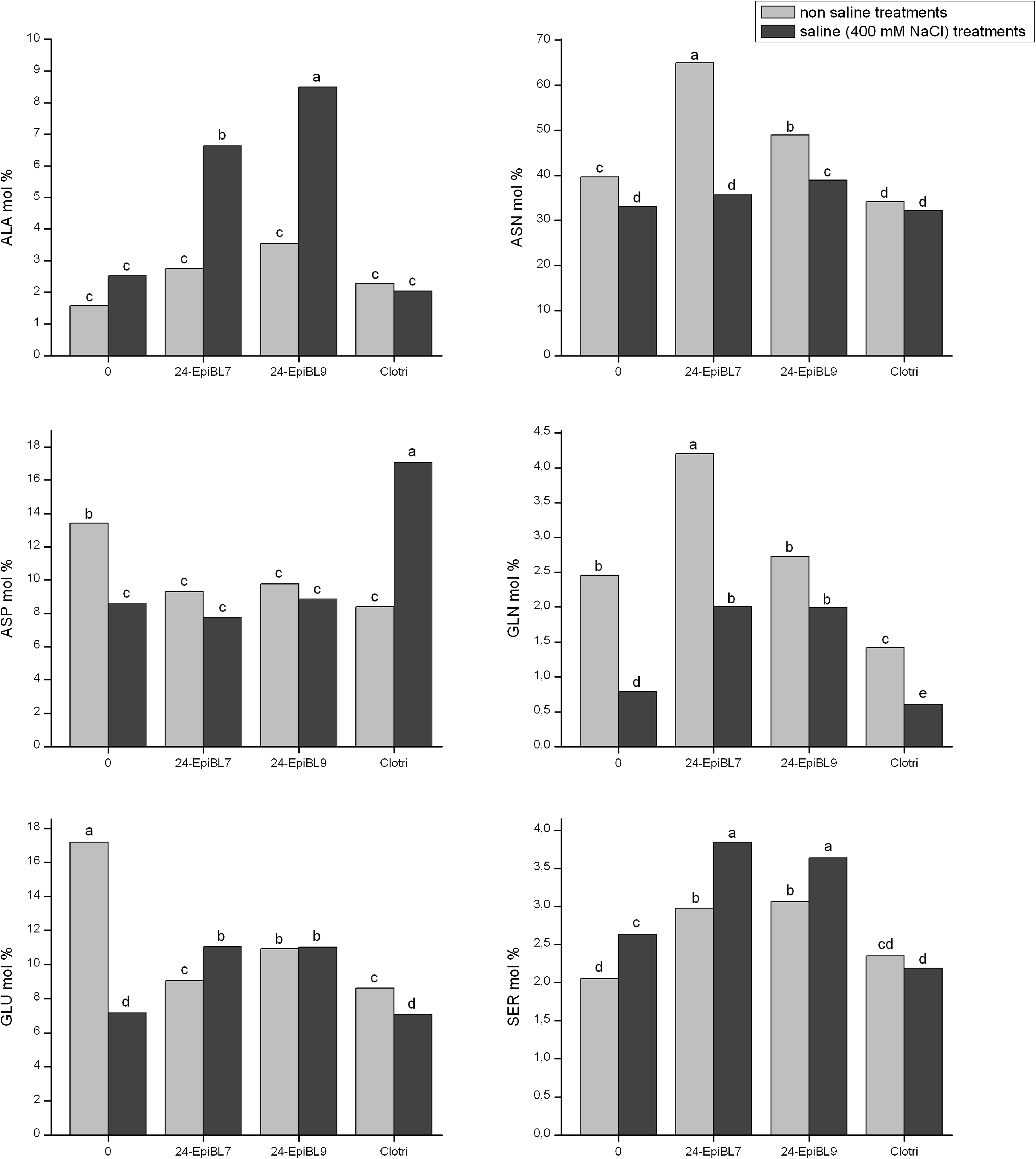

Supplement: Supplementary file 3 — Authors’ original file for figure 3 [file 40529_2012_8_MOESM3_ESM.tif]
